# Integrated transcriptome expression profiling reveals a novel lncRNA associated with L-DOPA-induced dyskinesia in a rat model of Parkinson’s disease

**DOI:** 10.18632/aging.102652

**Published:** 2020-01-10

**Authors:** Chun-Lei Han, Yun-Peng Liu, Yun-Peng Sui, Ning Chen, Ting-Ting Du, Ying Jiang, Chen-Jia Guo, Kai-Liang Wang, Qiao Wang, Shi-Ying Fan, Michitomo Shimabukuro, Fan-Gang Meng, Fang Yuan, Jian-Guo Zhang

**Affiliations:** 1 Department of Functional Neurosurgery, Beijing Neurosurgical Institute, Capital Medical University, Beijing, China; 2 Beijing Key Laboratory of Neurostimulation, Beijing, China; 3 Department of Neurosurgery, Beijing Tiantan Hospital, Capital Medical University, Beijing, China; 4 Department of Pathology, School of Basic Medical Sciences, Capital Medical University, Beijing, China; 5 Department of Pathophysiology, Beijing Neurosurgical Institute, Capital Medical University, Beijing, China

**Keywords:** Parkinson’s disease, levodopa-induced dyskinesia, long noncoding RNA, RNA sequencing

## Abstract

Levodopa-induced dyskinesia (LID) is a common complication of chronic dopamine replacement therapy in the treatment of Parkinson’s disease (PD). Long noncoding RNAs regulate gene expression and participate in many biological processes. However, the role of long noncoding RNAs in LID is not well understood. In the present study, we examined the lncRNA transcriptome profile of a rat model of PD and LID by RNA sequence and got a subset of lncRNAs, which were gradually decreased during the development of PD and LID. We further identified a previously uncharacterized long noncoding RNA, NONRATT023402.2, and its target genes glutathione S-transferase omega *(Gsto)*2 and prostaglandin E receptor (*Ptger*)3. All of them were decreased in the PD and LID rats as shown by quantitative real-time PCR, fluorescence *in situ* hybridization and western blotting. Pearson’s correlation analysis showed that their expression was positively correlated with the dyskinesia score of LID rats. *In vitro* experiments by small interfering RNA confirmed that slicing NONRATT023402 inhibited *Gsto2* and *Ptger3* and promoted the inflammatory response. These results demonstrate that NONRATT023402.2 may have inhibitive effects on the development of PD and LID.

## INTRODUCTION

Parkinson’s disease (PD) is the second most common neurodegenerative disorder after Alzheimer’s disease, and affects approximately 7 million people—mostly elderly—worldwide [1]. PD is characterized by motor symptoms, massive and selective loss of dopaminergic neurons in the substantia nigra (SN), and a decrease in striatal dopamine concentration [2] and is caused by genetic and non-genetic factors [3, 4]. Dopamine replacement therapy with the dopamine precursor. Levodopa (L-DOPA) is the most effective symptomatic treatment for PD. Although L-DOPA can significantly improve PD symptoms, long-term use typically leads to the gradual development of hyperkinetic involuntary movements known as L-DOPA-induced dyskinesia (LID), which manifests as nonrhythmic, nondirected involuntary movements that are unpredictable in onset and severity. LID is observed in nearly 90% of PD patients within approximately 10 years of initiating L-DOPA therapy [5]. To date, the molecular basis for LID are not fully understood; clarifying the molecular mechanisms is essential to identifying new therapeutic targets for its treatment [6, 7].

Long noncoding (lnc)RNAs are a type of ncRNA longer than 200 nucleotides [8] that regulate gene expression at the transcriptional, posttranscriptional, and epigenetic levels [9, 10]. LncRNAs containing micro (mi)RNA response elements can act as competing endogenous (ce)RNAs with mRNAs for shared target miRNAs [11]. LncRNAs play important roles in central nervous system development, neuronal function and maintenance, and neurodegenerative diseases including PD, and many studies employing high-throughput methods have demonstrated the dysregulation of lncRNAs in the brain [12, 13] and peripheral blood [14] of PD patients and in rodent [15] and cell [16, 17] models of PD. In fact, several special lncRNAs such as HAGLR opposite strand lncRNA [18], nuclear enriched abundant transcript 1 [19], antisense to Uchl1 [20], MAPT antisense RNA 1 [21], and metastasis-associated lung adenocarcinoma transcript 1 [22] have been implicated in the pathogenesis of PD.

Few studies to have have investigated the function of lncRNAs in LID pathogenesis. To address this point, in the present study we investigated the lncRNA profile of LID in a rat model.

## RESULTS

### Validation of the rat models of PD and LID

**Figure 1:**
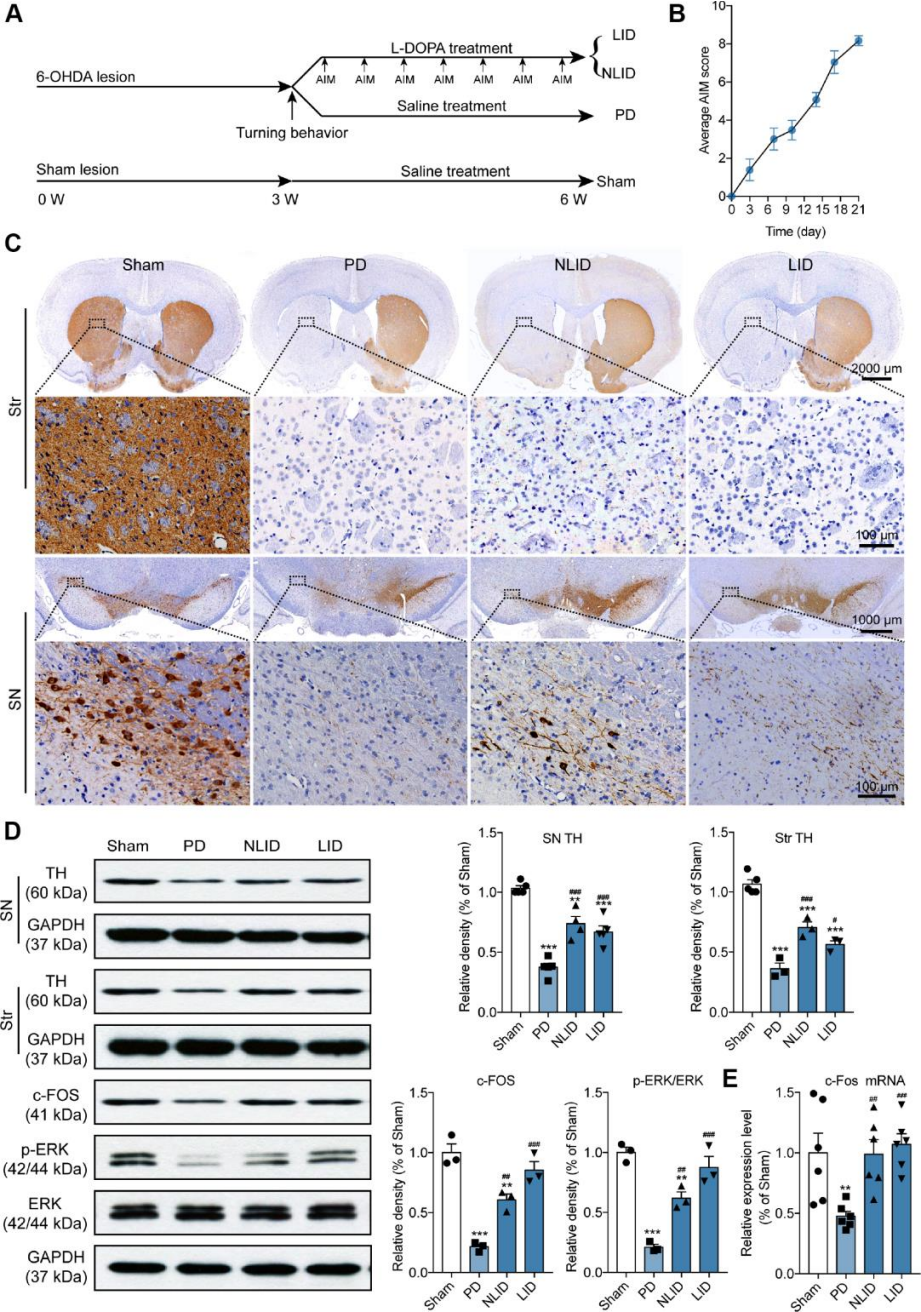
**Validation of the rat model of PD and LID.** (**A**) Experimental timeline showing 6-OHDA lesioning, L-DOPA administration, behavioral testing, and animal grouping. (**B**) Time course of the manifestation of AIMs scored every 3 days over a period of 21 days after the final L-DOPA administration (n = 15). (**C**) Representative photomicrographs of TH immunohistochemical staining in coronal brain sections of the striatum and SN of rats subjected to 6-OHDA injection into the right striatum (PD) with (LID) or without (NLID) L-DOPA administration. Magnified images correspond to labeled boxes in the upper panels (n = 3). (**D**) Quantification of TH expression in the striatum and SN and of c-FOS, p-ERK, and ERK expression in the striatum of PD and LID rats and their corresponding control groups (n = 3–5). The signal intensity of protein bands was normalized to that of GAPDH. (**E**) qRT-PCR detection of c-Fos expression in the striatum of PD and LID rats and their corresponding controls. Data are shown as mean ± SEM (n = 6). **P < 0.01, ***P < 0.001 vs. sham group; #P < 0.05, ##P < 0.01, ###P < 0.001 vs. PD group.

Rats were evaluated for a Parkinson-like phenotype 3 weeks after lesioning and animals with marked motor coordination deficits were selected for experiments (Figure 1A). All of the PD model rats showed more than seven contralateral turns per minute in the apomorphine-induced turning test 3 weeks after 6-Hydroxydopamine (6-OHDA) injection. Dyskinesia was quantified using a validated rating scale for abnormal involuntary movements (AIMs) [23]. Rats showing a high degree of LID with the average AIMs score more than 4 after chronic L-DOPA administration for 3 weeks were assigned to the LID group, whereas those with no apparent dyskinesia and the average AIMs score no more than 4 constituted the non-LID (NLID) group (Figure 1A and 1B). PD and non-PD rats treated with saline did not develop dyskinesia.

Immunohistochemical detection of tyrosine hydroxylase (TH), a marker of dopaminergic neurons [24], showed that striatal 6-OHDA injections resulted in a dramatic loss of dopaminergic neuron in the SN and dopaminergic neuron degeneration in the striatum of PD, LID, and NLID rats on the side ipsilateral to the injection site (Figure 1C). Western blot analysis of TH levels confirmed the results of TH immunohistochemistry: the protein level of TH in the striatum of PD, LID, and NLID rats was reduced relative to that in the sham-treated control group (Figure 1D). However, there was a slight increase in TH levels in the striatum of LID and NLID rats that were administered L-DOPA for 3 weeks compared with PD rats (Figure 1D), suggesting that L-DOPA prevents the loss of dopaminergic neurons.

Previous studies have shown that immediate-early genes (IEGs) such as c-Fos, FosB, and ΔFosB, are hallmarks of LID [7, 25], while extracellular signal-regulated kinase (ERK)1/2 signaling has been shown to be hyperactivated in LID models and patients [26, 27]. In this study, we confirmed that the rat model of LID was successfully established by assessing the expression of c-Fos and ERK1/2 in the striatum of LID rats. As expected, c-Fos protein (Figure 1D) and mRNA (Figure 1E) levels were decreased in PD rats compared with sham controls, but were increased in LID and NLID groups relative to PD model rats. The same trend was observed for phosphorylated (p-) ERK1/2 (Figure 1D), consistent with previous studies [28, 29]. These results indicate that PD and LID models were successfully established.

### lncRNA expression profiles of PD and LID rats

**Figure 2:**
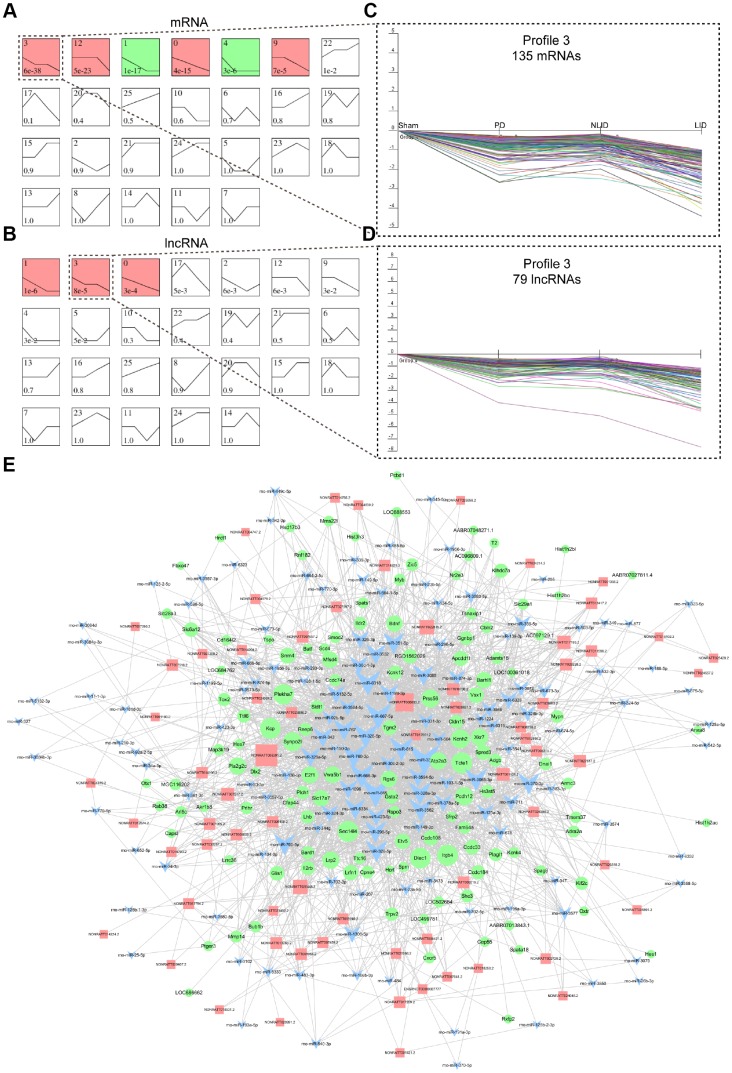
**LncRNA and mRNA expression signatures of PD and LID rats.** (**A**, **B**) Dynamic expression analyses of differentially expressed coding (**A**) and non-coding (**B**) genes. Differential expression patterns were determined based on 26 model profiles; each box represents a model expression profile, with the model profile number and P value shown in the box. Expression profiles with significant differences (P < 0.05) are indicated in red or green. (**C**, **D**) Profile 3 of mRNAs (**C**) and lncRNAs (**D**) in dotted boxes are shown in detail to the right. The horizontal axis shows the four groups (Sham, PD, NLID, and LID) and the vertical axis shows gene expression level. Each curve represents a single gene. (**E**) ceRNA network of lncRNAs with mRNAs in profile 3. LncRNAs, mRNAs, and miRNAs are represented by red squares, green circles, and blue triangles, respectively.

The lncRNAs and mRNAs that were differently expressed in PD and LID rats were screened by high-throughput RNA sequencing to determine the global lncRNA and mRNA landscape following 6-OHDA lesioning and L-DOPA administration. We summarized mRNA and lncRNA expression with 26 model profiles. Among the 26 expression profiles, six for mRNAs (Figure 2A) and three for lncRNAs (Figure 2B) showed significant P values (P < 0.05). Of these, Profile 3 contained 135 mRNAs (Table 1) and 79 lncRNAs (Table 2) that were decreased in PD rats compared to sham controls and further decreased in LID rats compared to NLID rats, indicating that these mRNAs and lncRNAs in profile 3 were closely related to PD and LID pathogenesis. Gene co-expression networks (Supplementary Figure 1A) and ceRNA networks (Figure 2C) were constructed to cluster the 79 lncRNAs and 135 coding mRNAs of profile 3, to determine the regulatory relationship between lncRNAs and mRNAs. Gene Ontology (GO) enrichment analysis of the potential target protein-coding genes of lncRNAs in profile 3 revealed biological processes that were enriched in oxidoreductase activity (GO terms: Oxidoreductase activity acting on CH-OH group of donors, Oxidoreductase activity acting on CH-OH group of donors, NAD or NADP as acceptor, and Oxidoreductase activity acting on NADPH), inflammatory response (GO terms: Regulation of interleukin-1 production, Positive regulation of tumor necrosis factor production, Chemokine receptor binding, Leukocyte-mediated cytotoxicity), neurotransmission (GO terms: Regulation of neurotransmitter secretion), and apoptosis (GO terms: Intrinsic apoptotic signaling pathway in response to endoplasmic reticulum stress and Activation of cysteine-type endopeptidase activity involved in apoptotic process), suggesting that the dysregulated lncRNAs are involved in these biological process (Supplementary Figure 1B).

**Table 1:** mRNAs in profile 3 of the gene expression analysis.

**Profile 3**				
AABR07005593.1	Ccdc184	Hist3h3	Mms22l	Slc17a7
AABR07013843.1	Ccdc33	Hrct1	Myb	Slc28a3
AABR07016578.1	Ccdc74a	Hs3st5	Mypn	Slc29a1
AABR07016845.1	Cd164l2	Hsd17b3	Nr2e3	Slc6a12
AABR07018064.1	Cep55	Il2rb	Otx1	Smoc2
AABR07027811.4	Cfap44	Ildr2	Oxtr	Spag8
AABR07048271.1	Cldn15	Itgb4	Pcbd1	Spata18
AC096809.1	Cpne4	Kcnh2	Pcdh12	Spats1
AC097129.1	Cxcr5	Kcnk12	Pla2g2c	Spred3
AC123253.2	Dlec1	Kcnk4	Plagl1	Sprn
Adamts18	Dlx2	Kcp	Plch1	Srrm4
Adgb	Dnai1	Kif2c	Plekha7	Synpo2l
Adra2a	E2f1	Klhdc7a	Prlhr	T2
Akr1b8	Etv5	Lhb	Prss56	Tcte1
Anxa8	Fam64a	LOC100361018	Ptger3	Tgm2
Apcdd1l	Fbxo47	LOC499781	Rab38	Tmem37
Arl5c	Ggnbp1	LOC502684	Reep6	Tox2
Armc3	Glis1	LOC684762	RGD1562029	Trpv2
Atp2a3	Gsto2	LOC686662	Rgs6	Tsnaxip1
Bard1	Hcrt	LOC688553	Rnf182	Tspo
Barhl1	Hes1	Lrfn1	Rspo3	Ttc16
Batf	Hes7	Lrp2	Rxfp2	Ttll6
Bdnf	Hist1h2ac	Lrrc36	Scd4	Vsx1
Bub1b	Hist1h2ac	Map3k19	Sec14l4	Vwa3b
Capsl	Hist1h2ao	Mfsd4	Sfrp2	Vwa5b1
Cbln2	Hist1h2bl	MGC116202	Shc3	Xkr7
Ccdc108	Hist1h2bo	Mmp14	Sidt1	Zic5

**Table 2:** LncRNAs in profile 3 of the gene expression analysis.

**Profile 3**		
NONRATT000216.2	NONRATT009993.2	NONRATT024024.2
NONRATT001131.2	NONRATT011596.2	NONRATT024046.2
NONRATT001180.2	NONRATT012417.2	NONRATT024214.2
NONRATT001358.2	NONRATT012674.2	NONRATT024557.2
NONRATT001421.2	NONRATT012936.2	NONRATT024630.2
NONRATT002729.2	NONRATT013263.2	NONRATT025429.2
NONRATT003319.2	NONRATT014106.2	NONRATT025956.2
NONRATT004126.2	NONRATT014204.2	NONRATT026386.2
NONRATT004376.2	NONRATT014224.2	NONRATT026498.2
NONRATT004419.2	NONRATT014766.2	NONRATT026518.2
NONRATT004747.2	NONRATT014929.2	NONRATT027617.2
NONRATT005391.2	NONRATT015102.2	NONRATT027997.2
NONRATT005891.2	NONRATT017193.2	NONRATT028621.2
NONRATT005964.2	NONRATT017209.2	NONRATT029228.2
NONRATT006053.2	NONRATT017794.2	NONRATT030267.2
NONRATT006238.2	NONRATT017951.2	ENSRNOT00000087777
NONRATT006431.2	NONRATT018250.2	MSTRG.15278.3
NONRATT007085.2	NONRATT018531.2	MSTRG.16299.3
NONRATT007290.2	NONRATT018736.2	MSTRG.17073.1
NONRATT007459.2	NONRATT019793.2	MSTRG.23728.9
NONRATT007516.2	NONRATT020448.2	MSTRG.23943.1
NONRATT007517.2	NONRATT020991.2	MSTRG.26499.20
NONRATT007641.2	NONRATT021380.2	MSTRG.38968.2
NONRATT007657.2	NONRATT021598.2	MSTRG.39805.1
NONRATT009240.2	NONRATT022815.2	MSTRG.40651.2
NONRATT009407.2	NONRATT023402.2	
NONRATT009800.2	NONRATT023886.2	

### Expression pattern of lncRNA NONRATT023402.2 and potential target genes

**Figure 3:**
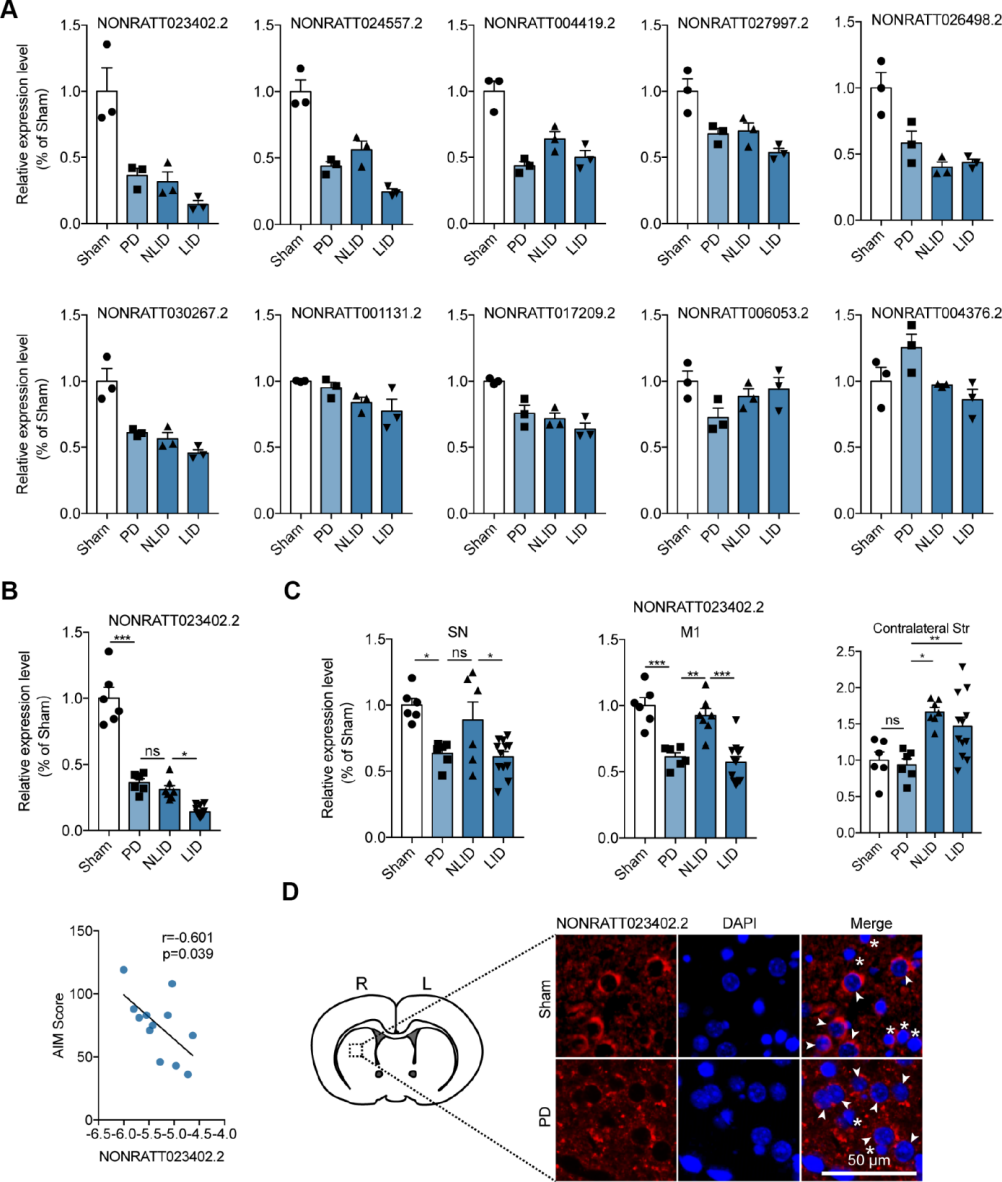
**Expression profiles of the lncRNA NONRATT023402.2.** (**A**) Validation of RNA sequencing results by qRT-PCR-based quantification of 10 lncRNAs from profile 3 (n = 3). (**B**) (Up) NONRATT023402.2 expression in the striatum of PD and LID rats and their corresponding control groups detected by qRT-PCR (n = 6–11). (Down) Pearson’s correlation coefficient between NONRATT023402.2 expression in the striatum of LID rats and AIM score (n = 11). (**C**) qRT-PCR analysis of NONRATT023402.2 levels in the SN, M1, and contralateral striatum of PD and LID rats and their corresponding controls (n = 6–11). (**D**) FISH labeling of NONRATT023402.2 in the striatum of rats. Arrows and asterisks indicate neurons and astrocytes, respectively. Data represent mean ± SEM. *P < 0.05, **P < 0.01, ***P < 0.001.

Ten lncRNAs in profile 3 were selected for validation according to the results of the gene co-expression network (Supplementary Figure 1A), ceRNA network (Figure 2C), and lncRNA target prediction. Their expressions in the striatum were analyzed by quantitative real-time (qRT-) PCR. Eight of the ten lncRNAs showed the same general expression trends as in profile 3, with the lncRNA NONRATT023402.2 being the most decreased (Figure 3A). The ceRNA network analysis showed that NONRATT023402.2 had multiple target genes (Supplementary Table 1), and a homology analysis showed that NONRATT023402.2 was highly conserved across human, mouse, and rats (Supplementary Table 2). We therefore focused on NONRATT023402.2 in subsequent experiments. The expression of lncRNA NONRATT023402.2 was decreased in PD rats compared to the sham group, and was further decreased in LID rats compared to NLID rats, but the differences in expression level between PD and NLID rats was not statistically significant (Figure 3B). A correlation analysis showed that NONRATT023402.2 level in the striatum was negatively correlated with the AIM score of LID rats (Figure 3B), indicating that the downregulation of NONRATT023402.2 in the striatum is associated with the development of PD and LID. Given that the SN and the primary motor cortex (M1) [30] as well as the contralateral striatum are also implicated in these disorders, NONRATT023402.2 expression in these areas was also evaluated by qRT-PCR. The levels in the SN and M1 areas were similar to that in the ipsilateral striatum, but the opposite trend was observed in the contralateral striatum (Figure 3C). Fluorescence *in situ* hybridization (FISH) analysis showed that NONRATT023402.2 was mainly localized in the cytoplasm of neurons (Figure 3D).

**Figure 4:**
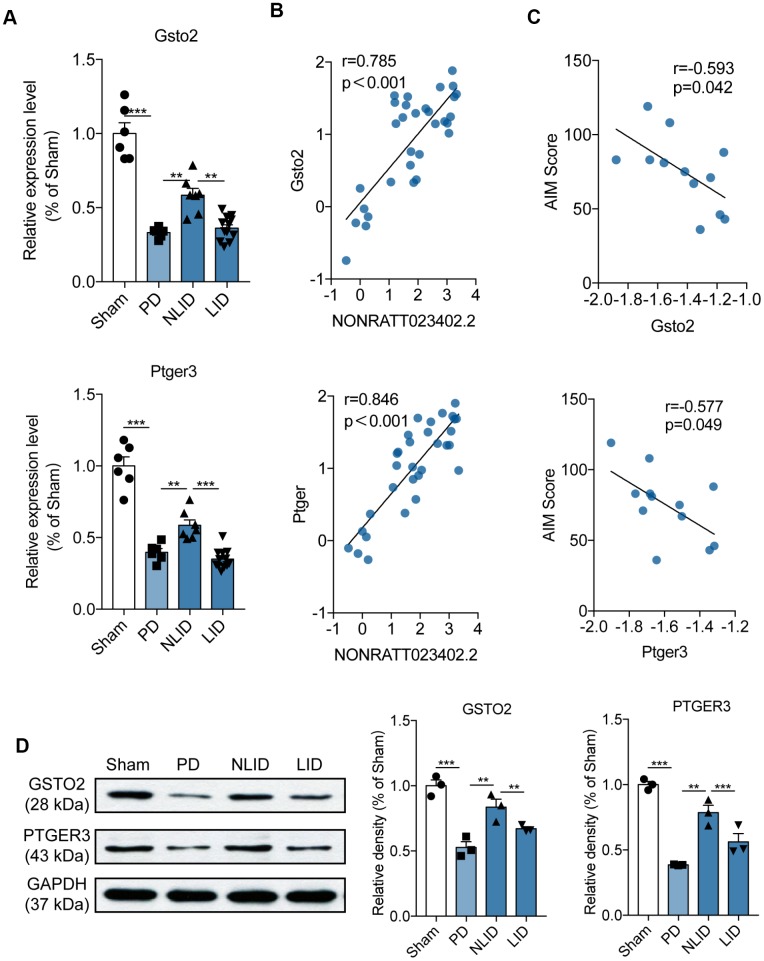
**Expression profiles of the potential target genes of lncRNA NONRATT023402.2.** (**A**) *Gsto2* and *Ptger3* expression determined by qRT-PCR in the striatum of PD and LID rats and their corresponding controls (n = 6–11). (**B**) Correlation between NONRATT023402.2 and *Gsto2* or *Ptger3* expression levels in the striatum of PD and LID rats and their corresponding controls (n = 11). (**C**) Correlation between *Gsto2* or *Ptger3* expression in the striatum of LID rats and AIM score (n = 11). (**D**). GSTO2 and PTGER3 protein levels in the striatum of PD and LID rats and their corresponding controls (n = 3), as determined by western blotting. The intensity of protein bands was quantified by densitometry and normalized to that of GAPDH. Data represent mean ± SEM. **P < 0.01, ***P < 0.001.

The ceRNA analysis identified 37 potential target protein-coding genes of NONRATT023402 (Supplementary Table 1). Five of these including glutathione S-transferase omega *(Gsto)*2, prostaglandin E receptor (*Ptger*)3, *potassium voltage-gated channel subfamily H member 2 (Kcnh2)*, *Map3k19*, and *solute carrier 28 family 28 member* (*Slc28*) *a3* were selected for qRT-PCR validation. *Gsto2* and *Ptger3* levels were altered in PD and LID rats (Figure 4A and Supplementary Figure 2)—i.e., they were decreased in PD rats after 6-OHDA administration and further reduced in LID rats, but these trends were reversed in NLID rats after L-DOPA administration (Figure 4A). The correlation analysis showed that both genes were negatively correlated with NONRATT023402.2 (Figure 4B) and with the AIM score of LID rats (Figure 4C). The protein levels of *GSTO2* and *PTGER3* were also quantitated by western blotting, which yielded results that were consistent with the mRNA expression (Figure 4D). These results indicate that downregulation of NONRATT023402.2 may contribute to the occurrence of PD and LID through positive regulation of *Gsto2* and *Ptger3*.

**Figure 5:**
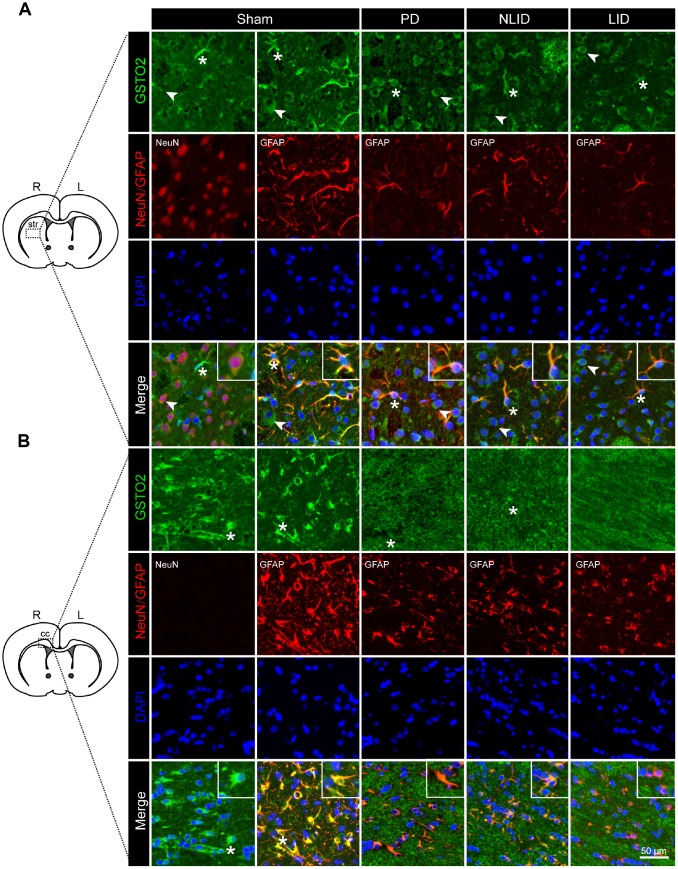
**Decreased expression of GSTO2 in neurons and astrocytes of the brain in PD and LID rats.** (**A**, **B**) Double immunofluorescence labeling of GSTO2 and neuron or astrocyte markers in the striatum (**A**) and corpus callosum (cc) (**B**) of PD and LID rats and their corresponding controls (n = 3). A single cell is shown in the insets. Arrows and asterisks indicate neurons and astrocytes, respectively, expressing GSTO2.

To determine the cellular localization of GSTO2 and PTGER3 proteins, we performed double immunofluorescence labeling of rat brain sections. GSTO2 was expressed in neurons and to a greater extent in astrocytes (Figure 5). Consistent with the results of qRT-PCR and western blot analyses, GSTO2 protein levels in both neurons and astrocytes were reduced after 6-OHDA lesioning and L-DOPA administration (Figure 5A). Immunofluorescence analysis revealed that GSTO2 was also expressed in astrocytes in the corpus callosum (cc) (Figure 5B). In contrast, PTGER3 was expressed only in neurons but showed the same decreasing trends, as determined by qRT-PCR and western blotting (Supplementary Figure 3). Thus, downregulation of NONRATT023402.2 contributes to the occurrence of PD and LID by regulating GSTO2 expression in neurons and astrocytes in the brain.

### Slicing lncRNA NONRATT023402.2 inhibits Gsto2 and Ptger3, and promotes inflammatory response *in vitro*

**Figure 6:**
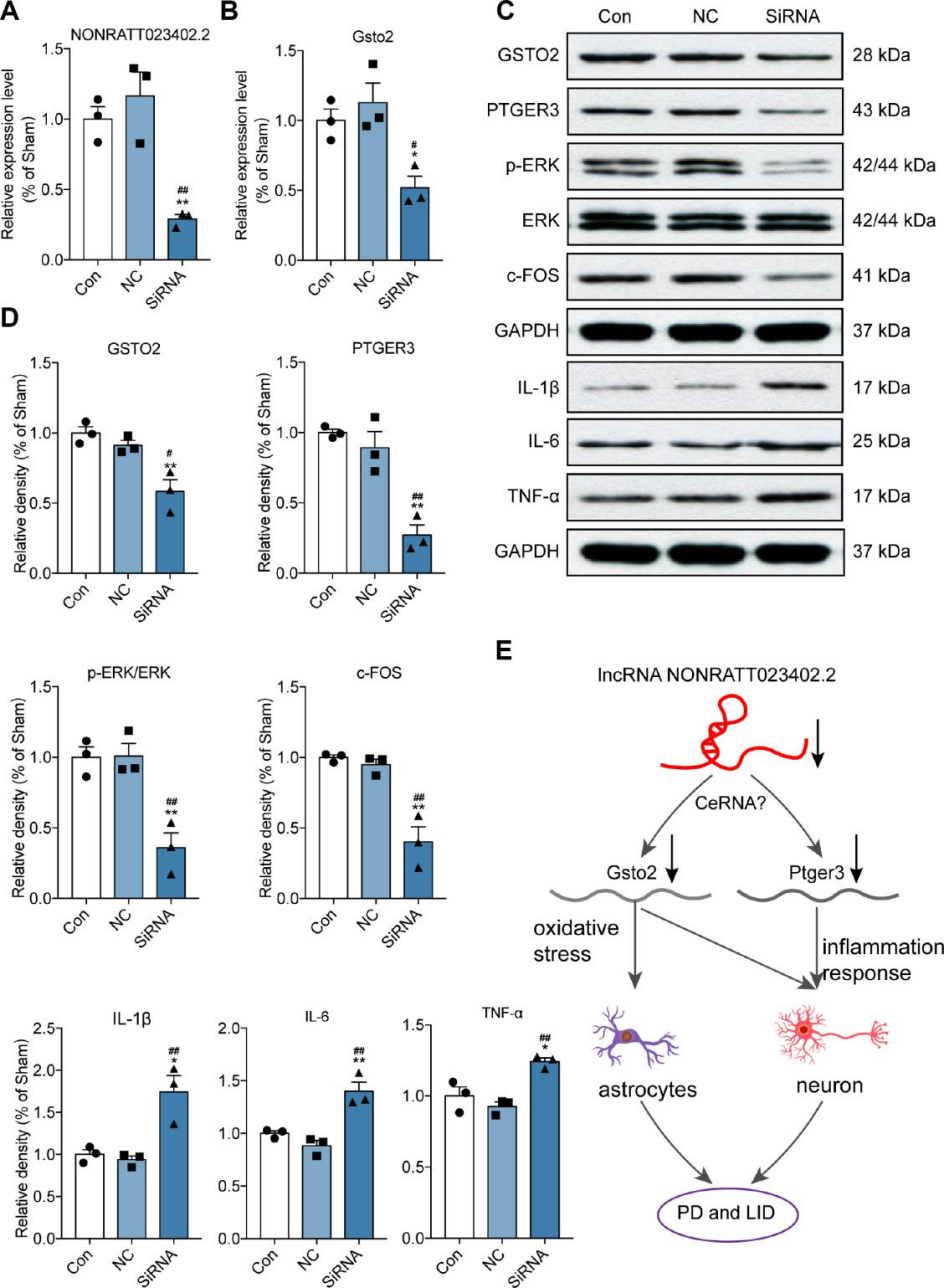
**Slicing lncRNA NONRATT023402.2 inhibited Gsto2 and Ptger3, and promoted inflammatory response *in vivo*.** (**A**, **B**) qRT-PCR detection of NONRATT023402.2 and *Gsto2* levels in rat PC12 cells transfected with NONRATT023402.2 siRNA (n = 3). (**C**, **D**) Protein levels of GSTO2, PTGER3, c-FOS, p-EERK, ERK, IL-1β, IL-6 and TNF-α in PC12 cells transfected with NONRATT023402.2 siRNA (n = 3), as determined by western blotting. The signal intensity of protein bands was quantified by densitometry and normalized to that of GAPDH. (**E**) Schematic representation of the regulatory mechanism of NONRATT023402.2 in PD and LID. Downregulation of NONRATT023402.2 leads to the decline of GSTO2 and PTGER3, possibly through a ceRNA-type mechanism. The decrease in GSTO2 results in increased oxidative stress in neurons and astrocytes, whereas the decrease in PTGER3 promotes inflammation in neurons; these ultimately contribute to the development of PD/LID. Data represent mean ± SEM. *P < 0.05, **P < 0.01 vs. control group; ^#^P < 0.05, ^##^P < 0.01 vs. negative control (NC) group.

To confirm the relationship between NONRATT023402.2 and its potential target genes *Gsto2* and *Ptger3*, PC12 cells were transfected with a small interfering (si)RNA targeting NONRATT023402. The qRT-PCR analysis showed that the siRNA inhibited the expression of NONRATT023402.2 as well as the mRNA level of *Gsto2* (Figure 6A and 6B) and protein levels of GSTO2 and PTGER3 (Figure 6C and 6D). We also examined the protein levels of the inflammatory factors IL-1β, IL-6, TNF-α and LID biomarkers. The results showed that NONRATT023402.2 knockdown promoted the expression of proinflammatory factors and inhibited the expression of c-FOS and the phosphorylation of ERK1/2, indicating that NONRATT023402.2 participates in the development of LID via *Gsto2* and *Ptger3* (Figure 6C and 6D). These results indicate that NONRATT023402.2 may participate the genesis of PD and LID through *activating* the expression of *Gsto2* and *Ptger3* genes and inhibiting the inflammatory response.

## DISCUSSION

In this study we examined lncRNA expression profiles in well-established rodent PD and LID models to identify lncRNAs that were dysregulated in these two disorders. We found that several lncRNAs including NONRATT023402 were decreased with the progression of PD and LID. We also identified target genes of NONRATT023402, *Gsto2* and *Ptger3*, that are presumably involved in the progression of PD and LID. *In vitro* studies using PC12 cells confirmed the regulatory relationship between NONRATT023402 and *Gsto2* and *Ptger3*.

6-OHDA is widely used to establish animal PD/LID models [31, 32], as it causes the degeneration of dopaminergic neurons in the ipsilateral striatum and SN, which is consistent with the pathologic changes observed in the brain of PD patients. The results of the western blot and immunohistochemical analyses also revealed significant pathologic changes in the striatum and SN, indicating the successful establishment of the PD and LID models. Three weeks of L-DOPA treatment resulted in AIM but prevented dopaminergic neuron degeneration, as evidenced by the high TH protein levels in LID and NLID rats. This is expected, as L-DOPA treatment was shown to have neuroprotective effects on dopaminergic neurons of in animal models of PD [28, 33–35].

IEGs are genes that are transiently and rapidly activated in response to a wide variety of stimuli, with about 40 identified to date. IEGs such as c-Fos, FosB, and ΔFosB [36–38], were shown to be increased in the striatum of LID animals [7], which was positively correlated with dyskinesia severity in a primate model of PD [25]. ERK signaling, which acts upstream of IEGs, is activated in LID [39, 40]; ERK1/2 phosphorylation was positively correlated with increased ΔFosB level and with the degree of dyskinesia in mice [26], whereas ERK inhibition reduced LID incidence and severity [27]. In order to validate our LID model, in this study we not only determined the AIM score but also examined protein and mRNA levels of c-Fos and ERK1/2 and found that they were reactivated in LID rats compared to PD rats, consistent with previous reports [36–40].

We compared the lncRNA and mRNA expression profiles of PD and LID rats and identified numerous dysregulated lncRNAs and mRNAs, demonstrating that both coding and noncoding genes are affected in these disorders [12–17]. A subset of lncRNAs and mRNAs showed the same trends during PD LID development—ie, they were decreased under 6-OHDA-induced PD and further decreased under L-DOPA-induced LID, with no changes in expression observed in NLID and PD rats. The expression patterns these genes suggested an association with the development of PD and LID. We performed target gene prediction for lncRNAs in profile 3 revealed mRNAs with functions related to oxidoreductase activity, inflammatory response, neurotransmission, and apoptosis, which were previously shown to be abnormal in PD and LID [41, 42].

NONRATT023402.2 is a novel lncRNA located on chromosome 5 with a length of 1290 bp. While highly conserved genes tend to have important functions in the maintenance of normal biological function in organisms, lncRNAs lack evolutionary conservation unlike coding genes, miRNAs, and circRNAs [43, 44]. Our homology analysis showed that the lncRNA NONRATT023402.2 is highly conserved across human, mouse, and rat, suggesting that is has an important effect in basic biological processes. LID involves many brain areas including the striatum, SN, and M1 [45]. We found here that NONRATT023402.2 expression was perturbed in various brain areas of PD/LID rats. However, the opposite trend was observed in the contralateral striatum, indicating a potential compensatory response. The FISH analysis showed that NONRATT023402.2 was expressed in neurons throughout the brain, suggesting that it functions in the maintenance of these cells. Meanwhile, its localization in the cytoplasm indicated that it might act as a ceRNA manner. Although we did not carry out *in vivo* experiments to investigate the function of NONRATT023402.2, a preliminary correlation analysis showed a negative correlation between the expression of NONRATT023402.2 and its target genes and dyskinesia score, highlighting the importance of NONRATT023402.2 in LID.

GSTO2 is an omega class glutathione S-transferase, which is a type of detoxifying enzyme in antioxidant systems that catalyzes the conjugation of reduced glutathione with various electrophiles and thereby eliminates both exogenous and endogenous toxic compounds [46, 47]. *Gsto* gene polymorphisms have been linked to PD risk and age at onset [48]. The protein encoded by *Ptger3*, EP3, is a G protein-coupled receptor and one of four known PGE2 receptors. EP3 has many biological functions associated with digestion, nervous system function, kidney reabsorption, and uterine contraction. *Ptger3* is abundantly expressed in the skin and PGE2-PTGER3 signaling has an anti-inflammatory function in allergic inflammation of the skin [49]. Additionally, PTGER3 signaling was shown to have inflammatory, amyloidogenic, and synaptotoxic effects in a mouse model of Alzheimer disease [50]. Thus, GSTO2 is associated with oxidative stress whereas PTGER3 is mainly involved in inflammation. In this study, the protein levels of GSTO2 in both neurons and astrocytes were decreased in PD and LID rats, whereas PTGER3 was expressed only in neurons and was decreased in the PD and LID model. Our *in vitro* results confirmed that Gsto2 and Ptger3 are activated by NONRATT023402.2. The degeneration of dopaminergic neurons in PD is related to mitochondrial dysfunction, inflammation, and oxidative stress whereas inflammation is observed in LID [51]. Astrocytes confer neuroprotection through the release of trophic factors and antioxidant molecules [52]. Therefore, the biological functions of GSTO2 and PTGER3 in the development of PD and LID warrant further study.

Based on our observations, we speculate that downregulation of the lncRNA NONRATT023402.2 in PD and LID leads to decreased GSTO2 and PTGER3 expression, which could constitute a ceRNA network. The decreases in GSTO2 and PTGER3 levels may reduce oxidative stress in neurons and astrocytes and inflammation in neurons, respectively, leading to the development of PD/LID (Figure 6E). These findings provide insight into the molecular mechanisms of PD/LID as well as novel therapeutic targets for the treatment of these disorders.

## MATERIALS AND METHODS

### Animals

Male Sprague-Dawley rats were obtained from Vital-River Experimental Animal Technology Co. (Beijing, China) and were maintained in a temperature-controlled room on a 12:12-h light/dark cycle with free access to standard food and water. Animal experiments were carried out according to the Chinese Animal Welfare Act and Guidance for Animal Experimentation of Capital Medical University. The study protocol was approved by the Ethics Committee of Beijing Neurosurgical Institute, Capital Medical University (protocol no. AEEI-2018-200).

### 6-OHDA lesioning, L-DOPA administration, and behavioral testing

Rats were unilaterally lesioned by injection of 6-OHDA (12 μg/2.4 μl; Sigma-Aldrich, St. Louis, MO, USA) into the medial forebrain bundle (3.6 mm posterior and 8.2 mm ventral to bregma and 1.8 mm lateral to the midline) using a Hamilton syringe after anesthetization with 2%–3% isoflurane through an animal anesthesia ventilator system (RWD Life Science Co., Shenzhen, China). The rate of injection was 0.5 μl/min and the syringe was left in place for an additional 5 min to allow diffusion of 6-OHDA before it was slowly retracted. Turning behavior was recorded 3 weeks postlesion over a 90-min period after injection of apomorphine (0.5 mg/kg by subcutaneous injection), and rats showing more than seven full, ipsilateral turns per minute were selected for L-DOPA administration.

Starting 3 days after the turning behavior test, rats received single daily intraperitoneal injections of methyl L-DOPA/benserazide (6 mg/kg; Sigma–Aldrich) for 21 days. 6-OHDA-lesioned control rats received single daily injections of the same volume of saline. Abnormal AIMs were scored every 3 days (eight times in total) according to the dyskinesia scale and rating criteria [23] for 3 h following L-DOPA injection. Rats showing a high degree of LID with the average AIMs score more than 4 after chronic L-DOPA administration for 3 weeks were assigned to the LID group, whereas those with no apparent dyskinesia and the average AIMs score no more than 4 constituted the NLID group (Figure 1A). Rats were sacrificed 6 weeks after 6-OHDA injection for analyses.

### Cell culture and treatments

Rat adrenal pheochromocytoma cells (PC12, well-differentiated) were cultured at 37 °C and 5% CO_2_ in Roswell Park Memorial Institute 1640 medium containing 10% fetal bovine serum (both from Gibco, Grand Island, NY, USA). For cell transfection, cells were seeded in a 6-well plate and treated with 2 ml of transfection mixture containing 12 μl riboFECT CP Reagent and 200 nM Smart Silencer siRNA or negative control siRNA (RiboBio, Guangzhou, China). Cells were collected 48 h after transfection for analysis.

### RNA extraction, library construction, and sequencing

Total RNA was isolated from the right striatum of rats using a RNeasy mini kit (Qiagen, Hilden, Germany) according to the manufacturer’s instructions. Strand-specific libraries were prepared using the TruSeq Stranded Total RNA Sample Preparation kit (Illumina, San Diego, CA, USA). Purified libraries were quantified with a Qubit 2.0 fluorometer (Life Technologies, Carlsbad, CA, USA), and an Agilent 2100 bioanalyzer (Agilent Technologies, Santa Clara, CA, USA) was used to confirm the insert size and calculate the molar concentration. The cluster was generated by cBot with the library diluted to 10 pM and then sequenced on the Illumina HiSeq X-ten system. Library construction and sequencing were performed by Shanghai Biotechnology Corp. (Shanghai, China).

### Analysis of expression data

Sequenced raw reads were preprocessed by filtering out rRNA reads, adapters, short fragments, and other low-quality reads. HISAT2 [53] was used to map the clean reads to the human GRCh38 reference genome with two mismatches. After genome mapping, Stringtie [54, 55] was used with reference annotation to generate fragments per kilobase of transcript per million mapped reads (FPKM) values for known gene models. Differentially expressed genes were identified using edgeR [56]. The significance threshold (P value) in multiple tests was set as the false discovery rate (FDR). Fold change was also estimated based on the FPKM in each sample. Differentially expressed genes were selected using filtering criteria of FDR ≤ 0.05 and fold change ≥ 2.

### LncRNA identification and expression analysis

Stringtie [54, 55] was used to assemble reads into transcripts. Novel transcripts were obtained by comparing all assembled transcript isoforms with known human protein-coding transcripts using gffcompare. Putative lncRNAs were identified as novel transcripts using the following filters: length ≥ 200 bp; number of exons ≥ 2; open reading frame ≤ 300 bp; no or weak protein-coding ability (coding potential calculator score < 0 [57], category normalized citation impact score < 0 [58], and no significant similarity with the Pfam database [59]). To generate a unique set of lncRNAs, gffcompare was used to integrate RNA sequencing derived lincRNAs with known lncRNAs previously annotated with NONCODE v.5.

### Analysis of gene expression dynamics

The STEM algorithm for gene expression dynamics was used to profile gene expression series and determine the most probable set of clusters generating the observed series [60]. This method considers the dynamic nature of gene expression profiles during clustering and identifies the number of distinct clusters. According to the probability of changes in signal density of genes under different conditions, we identified a set of unique expression patterns for our models. The raw expression values were converted to log2 ratios. We defined unique profiles using a strategy for clustering gene expression data for short time series. The expression model profiles were related to the actual or expected number of genes assigned to each profile.

### GO analyses

To determine the biological function of the identified genes, analysis of GO terms (http://www.geneontology.org) enrichment was performed using clusterProfiler, an R package tool for comparing biological themes across gene clusters. Fisher’s exact test and the P value were used for detection; the selection criteria for significant GO or pathway were P < 0.05.

### Gene network construction

We constructed coding-noncoding gene co-expression networks with the differentially expressed lncRNAs and mRNAs. Those with a Pearson correlation coefficient ≥ 0.99 were selected and used to construct a network in each of the groups using Cytoscape. The analyses were performed by Shanghai Biotechnology Corp. (Shanghai, China).

The lncRNA-miRNA-mRNA ceRNA network was constructed based on the relationships between lncRNAs, miRNAs, and mRNAs. We selected the most highly correlated mRNA/lncRNA pairs by setting the correlation threshold to the 99^th^ percentile of the corresponding overall correlation distribution. We used seed match analysis to restrict the above-selected triplets to those in which both the lncRNA and mRNA had at least one perfect 6mer seed match with the shared miRNA. The ceRNA network was constructed by integrating the results of statistical and seed match analyses.

### LncRNA target prediction

Different algorithms were used to identify the cis- and trans-regulatory target genes of dysregulated lncRNAs. The first algorithm was programmed for target genes in cis. LncRNAs and potential target genes were paired and visualized using the UCSC genome browser (http://genome.ucsc.edu/). Genes transcribed within a 10-kb fragment up- or downstream of potentially relevant lncRNAs were considered as cis targets. Another algorithm was based on mRNA sequence complementarity and RNA duplex energy prediction and predicted the effects of lncRNAs binding to complete mRNA molecules. BLAST software was used for the initial screening and RNAplex software was used to identify trans-acting targets [61].

### qRT-PCR

Total RNA was extracted using the Ultrapure RNA Kit (CWbio Co., Beijing, China) and reverse transcription was performed using the HiFi-MMLV cDNA First Strand Synthesis Kit (CWbio Co., Beijing, China) according to the manufacturer’s instructions. qRT-PCR was performed with UltraSYBR Mixture (CWbio Co., Beijing, China) in a 20-μl reaction composed of 10 μl UltraSYBR Mixture, 0.4 μl each primer (10 μM), 2 μl cDNA template, and 7.2 μl dH_2_O on an ABI Prism 7500 instrument (Applied Biosystems, Foster City, CA, USA) under the following conditions: 95°C for 10 min, followed by 40 cycles of 95°C for 15 s and 60°C for 60 s. The forward and reverse primer sequences were as follows: NONRATT023402, 5′-GGCTATTCATACAAAGTGGCAGTT-3′ and 5′-CGCTGAGTCTCGTGAGTCTG-3′; Gsto2, 5′-AATCCGTCATTGCGTGTGAGT-3′ and 5′-GCTACCAGACATTCCTTGCTTAAC-3′; Ptger3, 5′-TCACCACGGAGACGGCTAT-3′ and 5′-GGCGAACGGCGATTAGGAA-3′; mitogen-activated protein kinase (MAPK)1, 5′-TGGAGCTGGACGACTTAC-3′ and 5′-GACACCGACATCTGAACG-3′; c-Fos, 5′-GTCCGTCTCTAGTGCCAACTTTAT-3′ and 5′-GTCTTCACCACTCCCGCTCT-3′; and rat glyceraldehyde 3-phosphate dehydrogenase (GAPDH), 5′-TGGAGTCTACTGGCGTCTT-3′ and 5′-TGTCATATTTCTCGTGGTTCA-3′. Each sample was run in triplicate. PCR products were confirmed by melting curve analysis. Relative expression levels were normalized to that of GAPDH with the 2^−ΔΔCt^ method.

### Western blotting

Western blotting analysis was performed as previously described [62] using the following primary antibodies: rabbit polyclonal anti-TH (ab112, 1:200), rabbit polyclonal anti-glial fibrillary acidic protein (GFAP; ab7260, 1:1500), rabbit polyclonal anti-c-FOS (ab7963, 1:500), rabbit polyclonal anti-IL-1β (ab9722, 1:500), mouse monoclonal anti-IL6 (ab9324, 1:500), and rabbit polyclonal anti-TNF-α (ab6671, 1:500) (all from Abcam, Cambridge, MA, USA); rabbit monoclonal anti-ERK1/2 (#4695, 1:1000), rabbit monoclonal anti-p-ERK1/2 (#4377, 1:500) (both from Cell Signaling Technology, Danvers, MA, USA); and rabbit polyclonal anti-Gsto2 (14562-1-AP, 1:1000) and rabbit polyclonal anti-PTGER3 (14357-1-AP, 1:500) (both from Proteintech, Rosemont, IL, USA). Rabbit monoclonal anti-GAPDH antibody (Abcam; ab181602, 1:3000) was used for the loading control. Protein band density was quantified using an Epson V330 Photo scanner (Seiko Epson, Nagano, Japan) and Quantity One software (Bio-Rad, Hercules, CA, USA).

### Immunofluorescence and immunohistochemical analyses

Formalin-fixed, paraffin-embedded sections (4-μm thick) were dried, washed, permeabilized, blocked in 5% goat serum, and incubated overnight with antibodies against GSTO2 (14562-1-AP; 1:50) and PTGER3 (14357-1-AP; 1:100) (both from (Proteintech); and GFAP (Ab53554; 1:500) and neuronal nuclei (Ab1024224; 1:300) (both from Abcam). Immunolabeled sections were washed and incubated with goat secondary antibodies conjugated with Alexa Fluor 594 or Alexa Fluor 488 (Merck Biosciences, Nottingham, UK). Sections were mounted with medium containing 4′,6-diamidino-2-phenylindole (DAPI) (Vector Laboratories, Burlingame, CA, USA). An antibody against TH (Abcam; ab112, 1:700) was used for immunohistochemical detection of TH. The sections were scanned and digitized using Pannoramic MIDI (3D HISTECH, Budapest, Hungary) and the images were analyzed using Pannoramic Viewer software (3D HISTECH).

### FISH

Paraffin-embedded sections (4 μm thick) were deparaffinized, dehydrated, and treated with 1 M sodium thiocyanate. The sections were then digested in a pepsin solution, fixed in 4% formaldehyde, dehydrated by sequential immersion in 70%, 85%, and 100% ethanol, and air-dried. The sections were incubated with a digoxin (DIG)-labeled probe (5′-DIG-AGTAACGCTGAGTCTCGTGAGTCTGGTTCCAT-DIG-3′) to detect NONRATT023402.2, followed by incubation with a DyLight 594-conjugated IgG fraction (Abcam; ab96873) coupled with a monoclonal mouse anti-DIG antibody (Abcam; ab116590). Nuclei were counterstained with DAPI. The sections were scanned and analyzed as described above.

### Statistical analysis

Statistical analyses were performed using Prism 5 software (GraphPad, La Jolla, CA, USA). Data were compared with the Student’s t test (two groups) or by one-way analysis of variance followed by an appropriate multiple comparisons test (more than two groups). Data are expressed as mean ± SEM.

## Supplementary Material

Supplementary Figures

Supplementary Tables
