# Correction for: Integrated transcriptome expression profiling reveals a novel lncRNA associated with l-DOPA-induced dyskinesia in a rat model of Parkinson’s disease

**DOI:** 10.18632/aging.204596

**Published:** 2023-03-14

**Authors:** Chun-Lei Han, Yun-Peng Liu, Yun-Peng Sui, Ning Chen, Ting-Ting Du, Ying Jiang, Chen-Jia Guo, Kai-Liang Wang, Qiao Wang, Shi-Ying Fan, Michitomo Shimabukuro, Fan-Gang Meng, Fang Yuan, Jian-Guo Zhang

**Affiliations:** 1 Department of Functional Neurosurgery, Beijing Neurosurgical Institute, Capital Medical University, Beijing, China; 2 Beijing Key Laboratory of Neurostimulation, Beijing, China; 3 Department of Neurosurgery, Beijing Tiantan Hospital, Capital Medical University, Beijing, China; 4 Department of Pathology, School of Basic Medical Sciences, Capital Medical University, Beijing, China; 5 Department of Pathophysiology, Beijing Neurosurgical Institute, Capital Medical University, Beijing, China

**Keywords:** Parkinson’s disease, levodopa-induced dyskinesia, long noncoding RNA, RNA sequencing

**This article has been corrected:** The authors found that the Western blot image of c-Fos in the fifth row of **Figure 1D** was incorrect due to their unintentional reuse of the image for the protein TH bands in the third row of Figure 1D. They replaced the incorrect image with an image of c-Fos protein from the same experiment. The authors would also like to clarify the appearance of the same GAPDH images in **Figures 1D** and **4D** and updated the legend to **Figure 4** with the following sentence: "The same GAPDH image was intentionally used in **Figures 1D** and **4D** as the internal control in the same Western blot.” These corrections have no impact on the experimental outcome or conclusions.

The corrected **Figures 1** and **4** are shown below.

**Figure 1:**
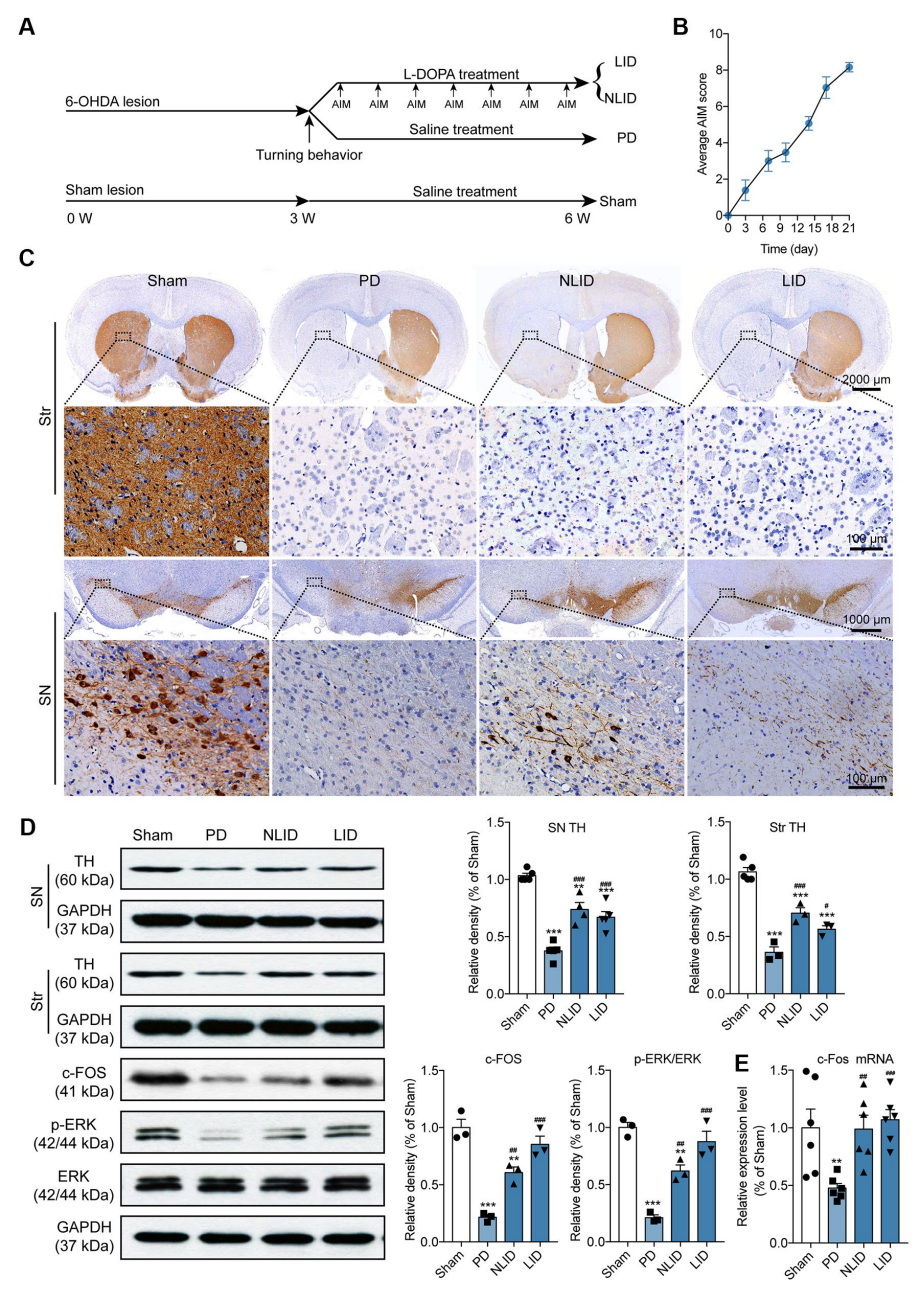
**Validation of the rat model of PD and LID.** (**A**) Experimental timeline showing 6-OHDA lesioning, l-DOPA administration, behavioral testing, and animal grouping. (**B**) Time course of the manifestation of AIMs scored every 3 days over a period of 21 days after the final l-DOPA administration (n = 15). (**C**) Representative photomicrographs of TH immunohistochemical staining in coronal brain sections of the striatum and SN of rats subjected to 6-OHDA injection into the right striatum (PD) with (LID) or without (NLID) l-DOPA administration. Magnified images correspond to labeled boxes in the upper panels (n = 3). (**D**) Quantification of TH expression in the striatum and SN and of c-FOS, p-ERK, and ERK expression in the striatum of PD and LID rats and their corresponding control groups (n = 3–5). The signal intensity of protein bands was normalized to that of GAPDH. (**E**) qRT-PCR detection of c-Fos expression in the striatum of PD and LID rats and their corresponding controls. Data are shown as mean ± SEM (n = 6). **P < 0.01, ***P < 0.001 vs. sham group; #P < 0.05, ##P < 0.01, ###P < 0.001 vs. PD group.

**Figure 4:**
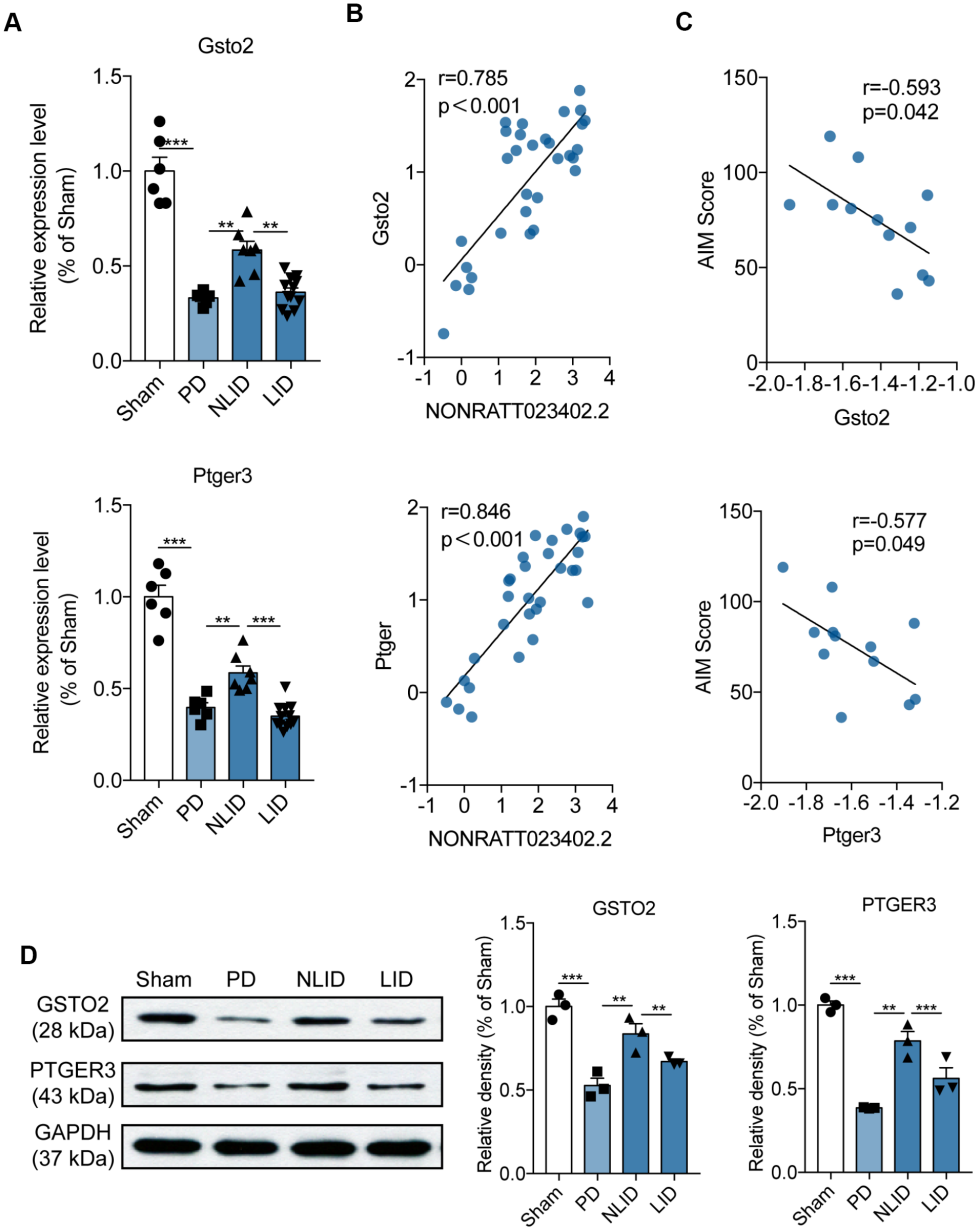
**Expression profiles of the potential target genes of lncRNA NONRATT023402.2.** (**A**) *Gsto2* and *Ptger3* expression determined by qRT-PCR in the striatum of PD and LID rats and their corresponding controls (n = 6–11). (**B**) Correlation between NONRATT023402.2 and *Gsto2* or *Ptger3* expression levels in the striatum of PD and LID rats and their corresponding controls (n = 11).  (**C**) Correlation between *Gsto2* or *Ptger3* expression in the striatum of LID rats and AIM score (n = 11). (**D**). GSTO2 and PTGER3 protein levels in the striatum of PD and LID rats and their corresponding controls (n = 3), as determined by western blotting. The intensity of protein bands was quantified by densitometry and normalized to that of GAPDH. The same GAPDH image was used intentionally in Figure 1D and Figure 4D as the internal control of the same Western blot. Data represent mean ± SEM. **P < 0.01, ***P < 0.001.

