# Supplementary material for: Integrated transcriptome expression profiling reveals a novel lncRNA associated with L-DOPA-induced dyskinesia in a rat model of Parkinson’s disease
Source: Aging (Albany NY). 2020 Jan 10;12(1):718–39. doi: 10.18632/aging.102652 (PMC6977703; doi:10.18632/aging.102652)
Supplement: Supplementary Figures [file aging-12-102652-s002..pdf]

SUPPLEMENTARY FIGURES

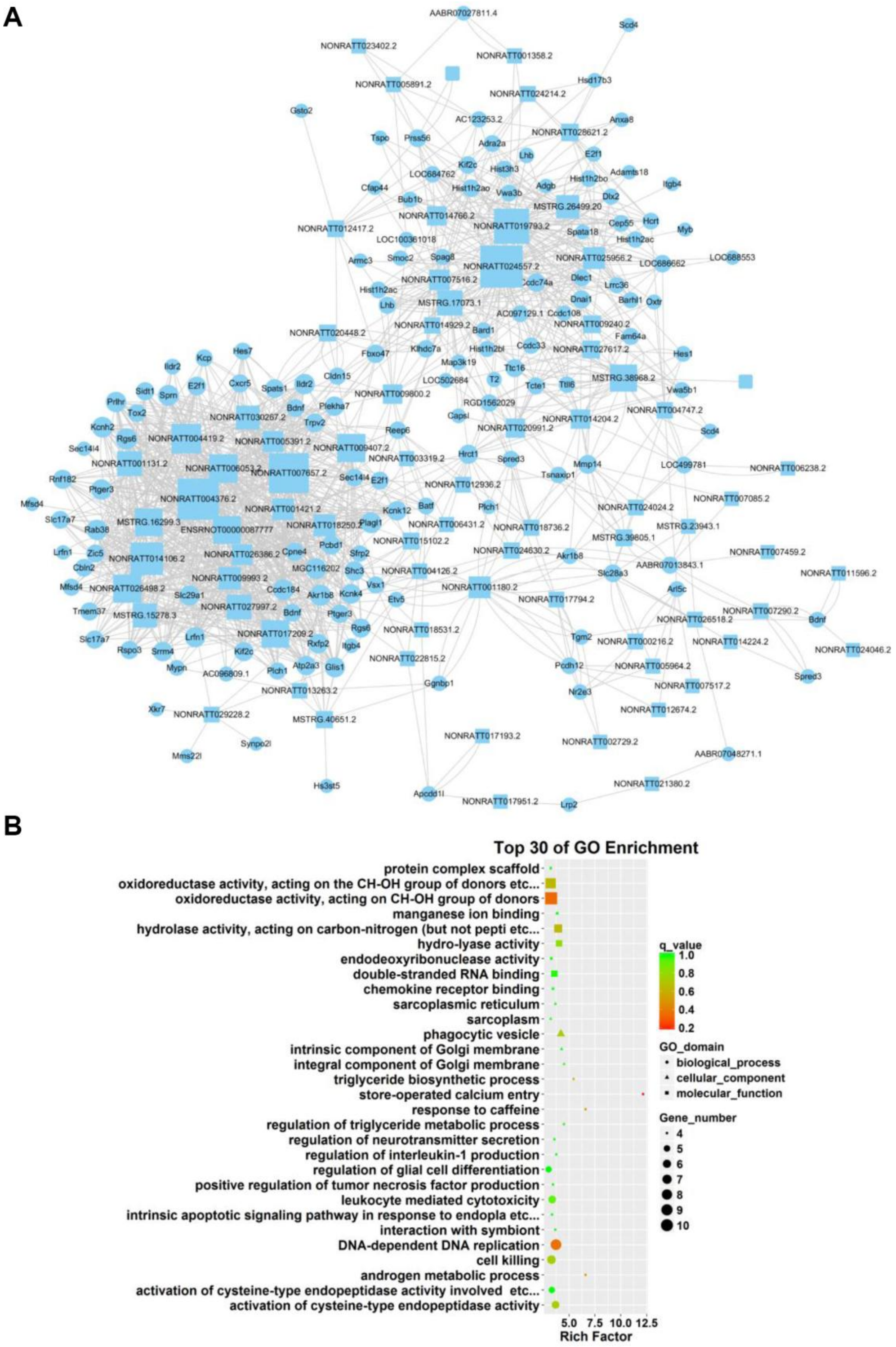

Supplementary Figure 1. Bioinformatics analysis of differentially expressed genes. (**A**) LncRNA and mRNA co-expression network consisting of lncRNAs and co-expressed protein-coding genes in profile 3. LncRNAs and protein-coding genes are represented by squares and circles, respectively. (**B**) GO pathway analysis of target genes of differentially expressed lncRNAs; both coding and noncoding RNAs were from profile 3. The top 30 GO pathways are listed.

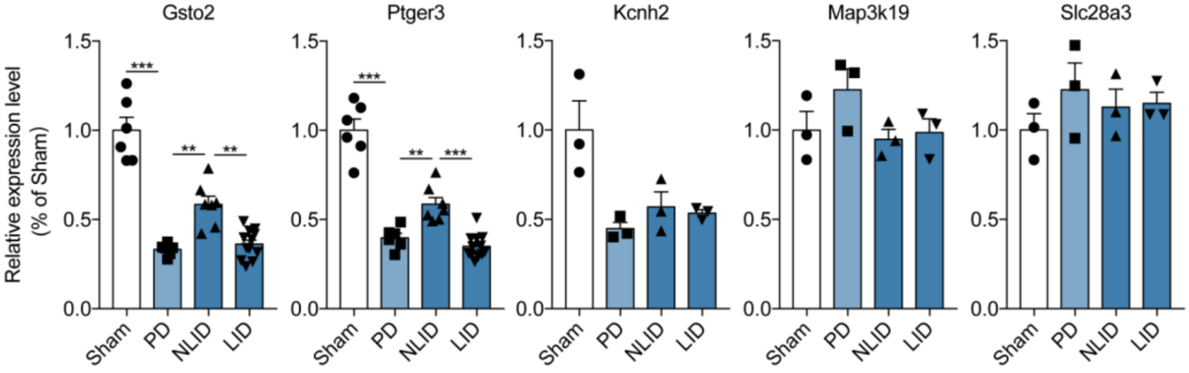

Supplementary Figure 2. Expression of target genes of the lncRNA NONRATT023402.2. qRT-PCR analysis of the expression of *Gsto2*, *Ptger3*, *Kcnh2*, *Map3k19*, and *Slc28a3* in the striatum of PD and LID rats and their corresponding controls. Data represent mean ± SEM (n = 3–11). **P < 0.01, ***P < 0.001.

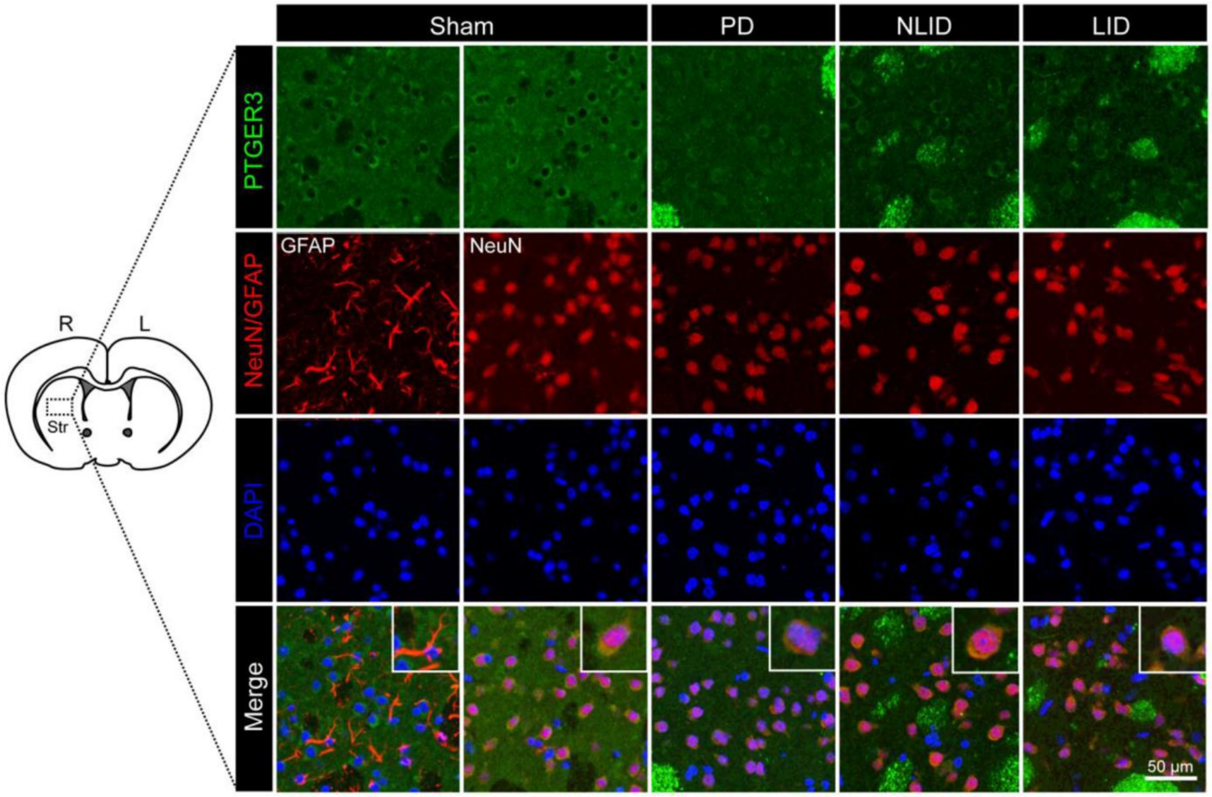

Supplementary Figure 3. PTGER3 protein expression is decreased in neurons in PD and LID rats. Double immunofluorescence labeling of GSTO2 and neuron or astrocyte markers in the striatum of PD and LID rats and their corresponding controls (n = 3). Single cells are shown in the inset.
