# Supplementary material for: Integrated transcriptome expression profiling reveals a novel lncRNA associated with L-DOPA-induced dyskinesia in a rat model of Parkinson’s disease
Source: Aging (Albany NY). 2020 Jan 10;12(1):718–39. doi: 10.18632/aging.102652 (PMC6977703; doi:10.18632/aging.102652)
Supplement: Supplementary Tables [file aging-12-102652-s001..pdf]

## SUPPLEMENTARY TABLES

**Supplementary Table 1. Candidate target genes of the lncRNA NONRATT023402.2 acting as a ceRNA.**

| LncRNA | miRNA | mRNA |
|---|---|---|
| NONRATT023402.2 | rno-miR-667-5p | Gsto2 |
| NONRATT023402.2 | rno-miR-667-5p | Ptger3 |
| NONRATT023402.2 | rno-miR-667-5p | Lrfn1 |
| NONRATT023402.2 | rno-miR-667-5p | Hrct1 |
| NONRATT023402.2 | rno-miR-667-5p | Dlx2 |
| NONRATT023402.2 | rno-miR-667-5p | Dlec1 |
| NONRATT023402.2 | rno-miR-667-5p | Sec14l4 |
| NONRATT023402.2 | rno-miR-667-5p | Lrrc36 |
| NONRATT023402.2 | rno-miR-667-5p | RGD1562029 |
| NONRATT023402.2 | rno-miR-667-5p | Prss56 |
| NONRATT023402.2 | rno-miR-667-5p | Synpo2l |
| NONRATT023402.2 | rno-miR-667-5p | Itgb4 |
| NONRATT023402.2 | rno-miR-667-5p | Kcnh2 |
| NONRATT023402.2 | rno-miR-667-5p | Map3k19 |
| NONRATT023402.2 | rno-miR-667-5p | Dnai1 |
| NONRATT023402.2 | rno-miR-667-5p | Xkr7 |
| NONRATT023402.2 | rno-miR-667-5p | Tox2 |
| NONRATT023402.2 | rno-miR-667-5p | Sidt1 |
| NONRATT023402.2 | rno-miR-667-5p | AC097129.1 |
| NONRATT023402.2 | rno-miR-667-5p | Lrp2 |
| NONRATT023402.2 | rno-miR-667-5p | LOC502684 |
| NONRATT023402.2 | rno-miR-667-5p | Slc28a3 |
| NONRATT023402.2 | rno-miR-1199-3p | Itgb4 |
| NONRATT023402.2 | rno-miR-1199-3p | Prlhr |
| NONRATT023402.2 | rno-miR-1199-3p | Bard1 |
| NONRATT023402.2 | rno-miR-1199-5p | Pcdh12 |
| NONRATT023402.2 | rno-miR-3584-5p | E2f1 |
| NONRATT023402.2 | rno-miR-3584-5p | LOC502684 |
| NONRATT023402.2 | rno-miR-3584-5p | Ccdc108 |
| NONRATT023402.2 | rno-miR-3584-5p | Plagl1 |
| NONRATT023402.2 | rno-miR-3584-5p | Lrp2 |
| NONRATT023402.2 | rno-miR-3584-5p | Bard1 |
| NONRATT023402.2 | rno-miR-3584-5p | Synpo2l |
| NONRATT023402.2 | rno-miR-3584-5p | Pcdh12 |
| NONRATT023402.2 | rno-miR-3584-5p | Kif2c |
| NONRATT023402.2 | rno-miR-3584-5p | Spats1 |
| NONRATT023402.2 | rno-miR-3584-5p | Mfsd4 |

**Supplementary Table 2. Homology analysis of lncRNA NONRATT023402.2 among human and mouse.**

| Species | lncRNA | lncRNA length (bp) | E-value | Score | Alignment | Identity | Identity percent (%) |
|---|---|---|---|---|---|---|---|
| Hsa | NONHSAT151711.1 | 829 | 1.00E-157 | 302 | 722 | 587 | 81.30 |
| | NONHSAT151710.1 | 632 | 1.00E-132 | 257 | 350 | 319 | 91.14 |
| | NONHSAT151712.1 | 838 | 9.00E-99 | 196 | 271 | 246 | 90.77 |
| Mmu | NONMMUT024326.2 | 624 | 0 | 476 | 628 | 579 | 92.20 |
| | NONMMUT040296.2 | 623 | 5.00E-137 | 265 | 650 | 529 | 81.38 |
| | NONMMUT048253.2 | 4840 | 4.00E-48 | 105 | 111 | 109 | 98.20 |
